# A Fatty Acids Mixture Reduces Anxiety-Like Behaviors in Infant Rats Mediated by GABA_A_ Receptors

**DOI:** 10.1155/2017/8798546

**Published:** 2017-12-17

**Authors:** Blandina Bernal-Morales, Jonathan Cueto-Escobedo, Gabriel Guillén-Ruiz, Juan F. Rodríguez-Landa, Carlos M. Contreras

**Affiliations:** ^1^Laboratorio de Neurofarmacología, Instituto de Neuroetología, Universidad Veracruzana, 91190 Xalapa, VER, Mexico; ^2^Unidad Periférica Xalapa, Instituto de Investigaciones Biomédicas, Universidad Nacional Autónoma de México, 91190 Xalapa, VER, Mexico

## Abstract

Fatty acids (C6–C18) found in human amniotic fluid, colostrum, and maternal milk reduce behavioral indicators of experimental anxiety in adult Wistar rats. Unknown, however, is whether the anxiolytic-like effects of fatty acids provide a natural mechanism against anxiety in young offspring. The present study assessed the anxiolytic-like effect of a mixture of lauric acid, myristic acid, palmitic acid, palmitoleic acid, stearic acid, oleic acid, elaidic acid, and linoleic acid in Wistar rats on postnatal day 28. Infant rats were subjected to the elevated plus maze, defensive burying test, and locomotor activity test. Diazepam was used as a reference anxiolytic drug. A group that was pretreated with picrotoxin was used to explore the participation of *γ*-aminobutyric acid-A (GABA_A_) receptors in the anxiolytic-like effects. Similar to diazepam, the fatty acid mixture significantly increased the frequency of entries into and time spent on the open arms of the elevated plus maze and decreased burying behavior in the defensive burying test, without producing significant changes in spontaneous locomotor activity. These anxiolytic-like effects were blocked by picrotoxin. Results suggest that these fatty acids that are contained in maternal fluid may reduce anxiety-like behavior by modulating GABAergic neurotransmission in infant 28-day-old rats.

## 1. Introduction

Amniotic fluid, colostrum, and milk provide water, proteins, fatty acids, and other biochemical products to offspring, mostly for nourishment and immune function. Maternal fluids also elicit behavioral effects that allow offspring to cope with stress. Amniotic fluid, colostrum, and milk in pigs* (Sus scrofa)* contain fatty acids that produce a calming effect in piglets, reflected by decreases in the number of fighting bouts and wounds [1]. In humans, detection of the odor of amniotic fluid reduces crying in newborns when the mother is absent [2]. Likewise, amniotic fluid and an artificial mixture of eight fatty acids (fatty acid mixture [FAM]) that are found in amniotic fluid produce orientating feeding responses [3]. Kindergarten children who were exposed to similar chemical compounds in their classroom exhibited relaxing and calming effects [1].

In preclinical studies, eight fatty acids (i.e., lauric acid, myristic acid, palmitic acid, palmitoleic acid, stearic acid, oleic acid, elaidic acid, and linoleic acid) that are contained in the aforementioned maternal fluids produced anxiolytic-like effects in adult rats in the elevated plus maze and defensive burying test [4, 5]. Similar to diazepam, amniotic fluid and the FAM also reduced the firing rate of lateral septal neurons that receive excitatory inputs from the medial amygdala [6], a neural circuit that regulates protective emotional responses to stress.

The effects of fatty acids on emotional responses have been scarcely studied in birds and mammals (e.g., infant humans, piglets, and adult cats and rats). Still unknown is whether anxiety is modifiable by a FAM in infant (postnatal day 28) Wistar rats through *γ*-aminobutyric acid (GABA) receptors. GABA_A_ receptors are the target of GABA receptor agonists (e.g., benzodiazepines), which increase channel opening and chloride influx, leading to anxiolytic-like effects in animals and therapeutic effects in humans [7]. The aim of the present study was to explore the potential anxiolytic-like effects of a FAM in 28-day-old Wistar rats using two validated models of experimental anxiety: elevated plus maze [8, 9] and defensive burying test [10, 11]. Diazepam was used as a reference anxiolytic drug. The noncompetitive GABA_A_ chloride ion channel antagonist picrotoxin was used to explore the possible participation of GABA_A_ receptors.

## 2. Methods

### 2.1. Ethics

This study was carried out in accordance with the recommendations of the* Guide for the Care and Use of Laboratory Animals* [12] and Mexican law requirements [13]. The experimental protocol was approved by the Biomedical Research Institute Ethical Committee of the National Autonomous University of México (UNAM).

### 2.2. Animals

The experiments included inbred Wistar rats that were obtained from the Biomedical Research Institute, UNAM. During gestation and after delivery, each of eight dams and their litters (standardized to 10 pups) remained individually allocated to acrylic boxes (44 cm length × 33 cm width × 20 cm height) under a 12 h/12 h light/dark cycle (lights on at 7:00 AM) with free access to water and food. The nest remained as undisturbed as possible, and the dams and pups were minimally handled when changing the bedding material (every 48 h). The offspring were weaned on postnatal day 21 and allocated to cages each with six to eight animals (44 cm length × 33 cm width × 20 cm height) following random assignment to the experimental groups. No more than three subjects from the same litter were included in the same experimental group. An equal number of male and female rats were included per group because no sexual dimorphism that is dependent on gonadal hormones has been observed in behavioral tests before puberty [14]. On the day of the tests (postnatal day 28), all of the infant animals remained in a holding area in the test room 1 h before the behavioral tests.

### 2.3. Elevated Plus Maze

The apparatus consisted of two opposite open and closed arms that were set in a plus configuration. The apparatus was elevated 50 cm above the floor in a test room that was illuminated at 40 lx. The dimensions of the closed arms were 50 cm length × 10 cm width, with 40 cm high walls. The dimensions of the open arms were 50 cm length × 10 cm width, with a short ledge to prevent the rats from falling. The open and closed arms intersected at a central area of 10 cm × 10 cm [15]. The test began by gently placing the rats in the center of the maze, facing an open arm. The time spent on the open arms and closed arms and total number of entries into the arms (open arms entries + closed arms entries) were quantified [9]. The percentage of open arm entries ([open entries]/[total entries] × 100 [16]) and Anxiety Index (AI = 1 − [([Open arm time/Test duration] + [Open arm entries/Total number of entries])/2] [17]) were calculated. In the same 5 min test, we evaluated the number of episodes of and time spent engaged in stretch-attend and head-dipping behaviors, which are considered measures of risk assessment [18]. “Stretch-attend” behavior was defined as any attempt to enter the open arms followed by an avoidance response, including the rat stretching forward and retracting to its original position. “Head-dipping” behavior was defined as the rat protruding its head over the ledge of an open arm and down toward the floor. After the elevated plus maze test, the rats were evaluated in the locomotor activity test. The device was cleaned with a 5% ethanol solution and dried with absorbent paper before each animal was placed in the apparatus.

### 2.4. Locomotor Activity Test

Each rat was placed in a locomotor activity monitor (Letica model LE 8811; Panlab S.L., Barcelona, Spain) that consisted of a black Perspex base (44 cm × 45 cm) with four transparent Perspex walls (36 cm height). Each test lasted 5 min. General locomotor activity (i.e., number of crossings, activity time [in seconds], and resting time [in seconds]) was automatically recorded by infrared detectors and software (Acti-Track v2.7.10; Panlab S.L., Barcelona, Spain). The software measured the number crossings as entries into each of the five zones of the floor, which was virtually divided using a virtual template. The tests were performed between 11:00 AM and 3:00 PM. The animals that were tested in the elevated plus maze were also tested in the locomotor activity test to discard or identify any possible motor effects of the treatments.

### 2.5. Defensive Burying Test

A round defensive burying chamber (19 cm diameter, 283.5 cm^2^ floor area) was previously validated for evaluating anxiety-like behavior in infant rats [19]. The chamber was placed in a sound-isolated box (55 cm × 40 cm × 46 cm; Coulbourn Instruments, Whitehall, PA, USA). Wood chips (Teklad Sani Chips 7990, 2 mm diameter, Harlan, Madison, WI, USA) covered the floor (2 cm depth) and were changed at the end of each test. The rats were individually and gently placed far from a metal prod (7 cm length) that was placed 1 cm above the wood chip surface. When the rat eventually touched the prod, it received a direct electrical current (0.1 mA) that was delivered by an electronic stimulator (Grass Instruments S44, Quincy, MA, USA) coupled in series to a stimulus isolation unit (SIU5, Grass Instruments, Quincy, MA, USA) and constant-current generator (CCUIA, Grass Instruments, Quincy, MA, USA). After the rat touched the prod, the latency to bury the prod and cumulative burying time were assessed in a 10 min session. For data interpretation, burying latency was considered an indicator of reactivity when an animal copes with stressful stimuli [20]. Cumulative burying time was considered an indicator of anxiety-like behavior [21, 22]. The locomotor activity data were automatically scored. Anxiety-like behavior was video-recorded and later scored by at least two observers. The statistical analysis of the data only included 99% agreement between the two independent observers.

### 2.6. Treatments

The FAM included fatty acids (analytical-grade, Sigma-Aldrich, St. Louis, MO, USA) based on the fatty acid concentrations that are measured in human amniotic fluid and produce anxiolytic-like effects [4]. The FAM consisted of lauric acid (4 *μ*g), myristic acid (30 *μ*g), palmitic acid (153 *μ*g), palmitoleic acid (71 *μ*g), stearic acid (37 *μ*g), oleic acid (80 *μ*g), elaidic acid (15 *μ*g), and linoleic acid (44 *μ*g) in 1 ml of vehicle. The rats were injected subcutaneously at 1 ml per rat.

The FAM was obtained by dissolving fatty acids in 100 ml of vehicle at a temperature < 40°C. The vehicle consisted of propylene 96% glycol and 4% ethanol (v1) and was injected subcutaneously at 1 ml per rat.

Diazepam (Valium 10, Hoffman-Roche, Basel, Switzerland) was used as the reference anxiolytic drug and injected intraperitoneally at a dose of 1 mg/kg dissolved in isotonic saline solution (v2) in a volume of 1 ml/300 g. This dose was selected because it produces anxiolytic-like effects in 28-day-old rats [19, 23, 24].

Analytical-grade picrotoxin (Sigma-Aldrich, St. Louis, MO, USA) was dissolved in v2 and injected intraperitoneally at 1 mg/kg in a volume of 1 ml/300 g. This dose blocks the anxiolytic-like effects of drugs, including fatty acids [4, 25].

Diazepam or the FAM was administered 1 h before the behavioral tests. Picrotoxin was administered 30 min before FAM administration based on the above studies. All rats were injected subcutaneously and intraperitoneally with corresponding treatments and vehicles.

### 2.7. Experimental Groups

To evaluate the effects of the FAM on anxiety-like behavior and participation of GABA_A_ receptors, rats were assigned to four experimental groups for testing in the elevated plus maze followed by the locomotor activity test: vehicle (v1 + v2, *n* = 8), FAM (*n* = 8), FAM + picrotoxin (*n* = 8), and diazepam (v1 + diazepam, *n* = 8). Other rats were submitted to the defensive burying test forming four groups: vehicle (v1 + v2, *n* = 8), FAM (v2 + FAM, *n* = 8), FAM + picrotoxin (*n* = 6), and diazepam (v1 + diazepam, *n* = 7).

To discard or identify any influence of EPM on locomotor activity test that could interfere in spontaneous locomotor activity, we tested two groups of 28-day-old rats. The first group injected with vehicles (i.p. and s.c., resp.) went to locomotor activity test before EPM (*n* = 6) and it was compared with the group injected with vehicles (i.p. and s.c., resp.) that was evaluated after EPM (*n* = 8).

### 2.8. Statistical Analysis

We used one-way analysis of variance (ANOVA) to analyze the effects of the treatments, followed by the Student-Newman-Keuls post hoc test. Values of *p* ≤ 0.05 were considered statistically significant. Comparison of locomotor activity in vehicle groups before and after EPM was analyzed by Student's *t*-test. The results are expressed as mean ± SEM.

## 3. Results

### 3.1. Elevated Plus Maze

**Figure 1:**
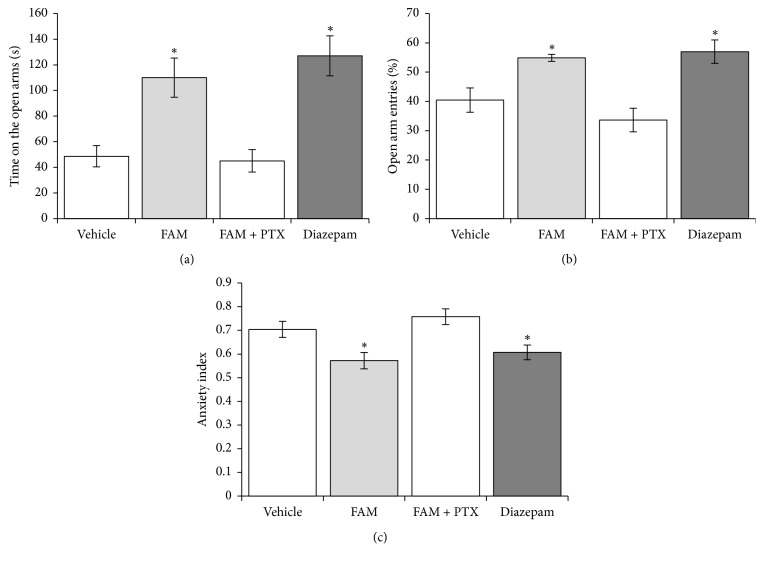
Elevated plus maze. The FAM and diazepam (a) increased the time spent on the open arms, (b) increased the percentage of entries into the open arms, and (c) decreased the Anxiety Index. Picrotoxin blocked the effects of the FAM. ^*∗*^*p* < 0.05, compared with vehicle and FAM + picrotoxin groups (Student-Newman-Keuls test). FAM, fatty acid mixture; PTX, picrotoxin.

A significant effect of the pharmacological treatments on time spent on the open arms was observed (*F*_3,28_ = 11.359, *p* < 0.001). The post hoc analysis revealed a longer time spent on the open arms in the FAM and diazepam groups compared with the vehicle and FAM + picrotoxin groups (*p* < 0.05; Figure 1(a)). The treatments significantly affected the percentage of open arm entries (*F*_3,28_ = 10.154, *p* < 0.001) and AI (*F*_3,28_ = 6.734, *p* < 0.001). Similar to diazepam, the FAM significantly increased the percentage of open arm entries and reduced the AI compared with the vehicle and FAM + picrotoxin groups (*p* < 0.05; Figures 1(b) and 1(c)).

The FAM did not significantly alter the number of arm entries or risk assessment behavior compared with the vehicle group. Diazepam significantly increased the number of open arm entries (*F*_3,28_ = 16.139, *p* < 0.001), closed arm entries (*F*_3,28_ = 4.522, *p* < 0.01), and total entries (*F*_3,28_ = 14.013, *p* < 0.001) and increased the time spent engaged in head-dipping behavior (*F*_3,28_ = 6.976, *p* < 0.001) but did not increase the time spent engaged in stretch-attend behavior (*F*_3,28_ = 0.191, *p* = 0.902) compared with the vehicle group (Table 1).

### 3.2. Locomotor Activity Test

Student's *t*-test revealed no significant differences in locomotor activity variables evaluated before or after EPM: number of crossings [*t*(12) = 3.917, *p* < 0.695] before 36.33 ± 7.5 versus after 40.2 ± 6.2]; total activity [*t*(12) = −1.832, *p* < 0.092] before 114.3 ± 10.5 s versus after 93.9 ± 5.5 s; and resting time [*t*(12) = 1.836, *p* < 0.091] before 185.5 ± 10.6 s versus after 206.0 ± 5.5 s. Therefore locomotor activity can be tested after EPM.

The FAM did not affect locomotor activity (Table 2). Diazepam produced a nonsignificant increase in the number of crossings (*F*_3,28_ = 2.859, *p* = 0.055). Diazepam decreased resting time (*F*_3,28_ = 9.225, *p* < 0.001) and increased total activity (*F*_3,28_ = 9.332, *p* < 0.001) compared with the vehicle, FAM, and FAM + picrotoxin groups.

### 3.3. Defensive Burying Test

**Figure 2:**
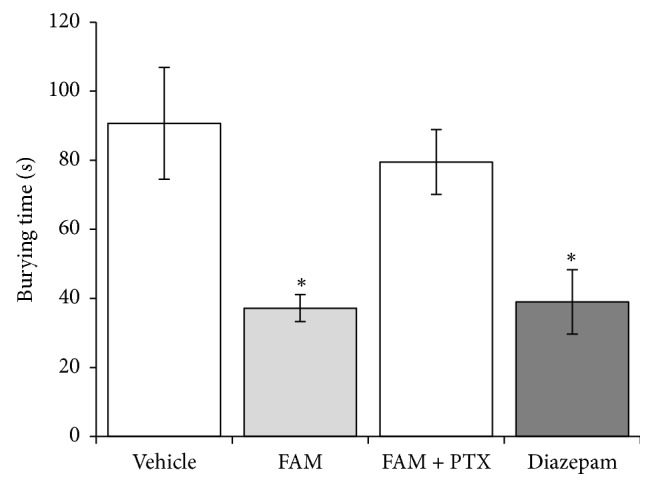
Defensive burying test. The FAM and diazepam decreased cumulative burying time compared with the vehicle and FAM + picrotoxin groups. ^*∗*^*p* < 0.05 (Student-Newman-Keuls test). FAM, fatty acid mixture; PTX, picrotoxin.

A trend toward increase in burying latency (*F*_3,28_ = 2.918, *p* = 0.054) was observed in the FAM (207.6 ± 55.2 s) and diazepam (116.3 ± 42.1 s) groups compared with the vehicle (62.6 ± 9.7 s) and FAM + picrotoxin (92.6 ± 20.3 s) groups. Cumulative burying time was significantly different between groups (*F*_3,28_ = 6.458, *p* < 0.002). The FAM and diazepam groups had an approximately 50% shorter cumulative burying time than the vehicle group, and picrotoxin blocked the effects of the FAM (Figure 2).

## 4. Discussion

The present study explored the anxiolytic-like effects of a FAM in 28-day-old Wistar rats in the elevated plus maze and defensive burying test. The FAM produced an anxiolytic-like effect in both behavioral tests, without altering spontaneous locomotor activity. This effect was blocked by pretreatment with picrotoxin, suggesting the participation of GABA_A_ receptors in these effects.

The elevated plus maze is a validated model for measuring anxiogenic- and anxiolytic-like effects in adult rats [9]. This model explores rodents' innate behavior that consists of aversion to open illuminated spaces and heights. Anxiolytic drugs increase the number of entries into and time spent on the open arms [8, 9] and increase risk assessment behavior, including stretch-attend and head-dipping behavior [18]. The model has also been successfully used in young rats [26–29]. In the present study, similar to diazepam, the FAM produced anxiolytic effects in infant rats.

In the defensive burying test, burying latency is an indicator of the animal's reactivity to the aversive shock-generating prod. Short burying latencies and long burying times are indicators of an anxiety-like state. Anxiolytic drugs increase burying latency and decrease cumulative burying [11, 30, 31]. Treatment with the FAM produced a longer burying latency and shorter cumulative burying compared with the vehicle group, illustrating an anxiolytic action of the FAM that is similar to diazepam.

Alterations in spontaneous locomotor activity that may be caused by pharmacological treatments must be tested when the elevated plus maze and defensive burying test are performed to discard possible locomotor actions. Anxiolytic doses of some drugs (e.g., 1.0–2.0 mg/kg diazepam) do not influence locomotor activity in adult rats [4, 10, 32]. In the present study, locomotor activity was assessed after the elevated plus maze test to exclude possible locomotor effects of the FAM. The lack of an effect of the FAM on (1) the number of crossings and other variables in the locomotor activity test and (2) total arm entries and number of entries into close arms in the elevated plus maze discards the possibility that the FAM-induced reduction of anxiety-like behavior was attributable to motor effects.

With regard to the mechanism of action of the FAM, previous studies suggested immediate actions of the FAM at the membrane level. The neuroprotective effects of fatty acids after systemic administration [33] occur through the lipocalin superfamily of protein transporters [34] that are found in human amniotic fluid [35], colostrum [36], and olfactory epithelia [37] that are connected to temporal brain structures that are related to emotional processing [38]. These effects suggest a mechanism of action at the neuronal membrane level because the fatty acids act on the phospholipid layer, resulting in tension and changes in conformation and conduction in ion channels [39]. Our research group found that the administration of a FAM and amniotic fluid in adult male rats decreased the firing rate of neurons in the lateral septal nucleus, which is connected to the medial amygdala. These effects were similar to those of diazepam [6]. The fatty acids that are contained in amniotic fluid may exert actions on brain structures that regulate emotional behavior, thus producing anxiolytic actions.

Some fatty acids (i.e., oleic acid, linoleic acid, ricinoleic acid, and arachidonic acid) modulate chloride ion channels [40], suggesting a possible mechanism of anxiolytic action of FAM in infant rats. In fact, changes in GABA_A_ receptor neurotransmission produce hyperpolarization that depends on the opening frequency of chloride ion channels [39, 40], and chloride ion channels participate in the anxiolytic-like actions of some endogenous steroids, such as progesterone and allopregnanolone [41–43]. These endogenous steroids at physiological concentrations promote channel opening frequency and increase chloride flux in the GABA system [43–45]. This action is involved in the therapeutic properties of diazepam, flunitrazepam, muscimol, and pentobarbital, which can be blocked by the noncompetitive GABA receptor antagonist picrotoxin [41, 46]. The anxiolytic effects of the FAM in adult rats appear to be mediated by GABA_A_ chloride channels. Indeed, picrotoxin but not bicuculline or flumazenil blocked the anxiolytic-like effect of the FAM on defensive burying and behavior in the elevated plus maze [25]. The anxiolytic-like effect of the FAM was blocked by picrotoxin in infant rats, thus confirming a mechanism of action that is similar to neurosteroids, benzodiazepines, barbiturates, and psychoactive drugs and involves GABA_A_ receptor chloride ion channels.

GABA_A_ receptors are functional at early stages of development, which corroborates the involvement of the GABA system in the mechanism of action of the FAM in infant rats. Ultrasonic vocalizations can be considered anxiety-like behavior. In 3- to 12-day-old rats, diazepam reduced ultrasonic vocalizations [23]. GABA reduces the amplitude and increases the latency in action potentials on isolated spinal cord preparation from 13-day-old rats. These effects are blocked by bicuculline and picrotoxin [47]. After birth, parental care has been shown to have protective effects against stress in offspring by reducing emotional fear responses and anxiety through the epigenetic regulation of GABA_A_ receptors in rat pups [48, 49] and humans [50–52]. On postnatal day 28, GABA_A_ receptors can functionally respond to anxiolytics, which was demonstrated in the behavioral tests in the present study. One limitation of the present study is that we did not measure GABA_A_ receptor conductance after FAM administration. Further biochemical studies are needed to clarify this issue. Nonetheless, the present results demonstrate that the FAM has neurobehavioral effects beyond simply providing nutritional needs.

Finally, the anxiolytic effect of subcutaneous FAM and their actions on GABA_A_ receptors in 28-day-old rats have been identified. Present study cannot discard some effects beyond the central nervous system, as other authors have reported on microbiota [53], glucose homeostasis [54], and skin [55] among others. However, possible peripheral effects that do not prevent anxiolytic effects observed in present study could be discarded with intracerebral microinjection of FAM in further experiments.

## 5. Conclusion

Fatty acids, such as lauric acid, myristic acid, palmitic acid, palmitoleic acid, stearic acid, oleic acid, elaidic acid, and linoleic acid, that are present in amniotic fluid, colostrum, and maternal milk reduce anxiety-like behavior in early postnatal life of rats through modulation of the GABAergic neurotransmission system.

## Figures and Tables

**Table 1:** Effects of the fatty acid mixture on behavior in the elevated plus maze in 28-day-old rats.

	Vehicle	FAM	FAM + picrotoxin	Diazepam	*p*
Open arm entries (*n*)	7.0 ± 1.2	10.5 ± 1.5	5.2 ± 1.3	19.8 ± 2.1^*∗*^	<0.001
Close arm entries (*n*)	10.0 ± 1.4	8.5 ± 1.0	9.6 ± 0.9	14.8 ± 1.6^*∗*^	<0.01
Total entries (*n*)	17.0 ± 2.3	19.0 ± 2.6	14.8 ± 1.8	34.7 ± 2.7^*∗*^	<0.001
Head-dipping (*s*)	5.3 ± 1.5	12.9 ± 3.6	4.9 ± 1.3	20.5 ± 3.6^*∗*^	<0.001
Stretch-attend (*s*)	13.0 ± 3.1	17.2 ± 3.9	12.9 ± 3.1	11.0 ± 3.5	0.902

FAM, fatty acid mixture. The data are expressed as mean ± SEM. ^*∗*^*p* < 0.05, versus all groups (Student-Newman-Keuls test).

**Table 2:** Effects of the fatty acid mixture on locomotor activity in 28-day-old rats.

	Vehicle	FAM	FAM + picrotoxin	Diazepam	*p*
Crossings (*n*)	40.2 ± 6.2	38.6 ± 8.0	36.8 ± 5.4	59.0 ± 3.7	0.055
Total activity (*s*)	93.9 ± 5.5	86.05 ± 12.36	78.4 ± 10.3	150.3 ± 12.9^*∗*^	<0.001
Resting time (*s*)	206.0 ± 5.5	214.0 ± 12.3	221.6 ± 10.2	148.0 ± 14.1^*∗*^	<0.001

FAM, fatty acid mixture. The data are expressed as mean ± SEM. ^*∗*^*p* < 0.05, versus all groups (Student-Newman-Keuls test).
